# Physiologic and Therapeutic Action of Thorium Derivatives

**Published:** 1913-10

**Authors:** 


PHYSIOLOGIC AND THERAPEUTIC ACTION OF THORIUM DERIVATIVES. De Nobele of Gand, *Arch. d'Elect. Med.,* July 10, 1913. Berzelius discovered thorium oxid in 1828, and, shortly after, isolated the metal. The minerals richest in thorium are monazite, a sand found in Brazil along certain rivers directly tributary to the ocean, thorite and thorianite. Hahn first extracted considerable quantities of radioactive derivatives of thorium from the residue of a mantle factory, in the form of the nitrate which is still the salt most used industrially. The pure salts of thorium are white, neither fluorescent nor phosphorescent, but show a marked tendency to pass into the colloid state. All salts of thorium are precipitated as oxalates. The oxid, formed by calcining the oxalate, is a white amorphous powder, having the power of condensing gases on its surface. The Curies discovered that certain thorium minerals emitted rays analogous to those of uranium and radium. There is somewhat contradictory testimony as to whether these rays are due to thorium itself, but Leslie has recently apparently proved that it does produce alpha rays, of very feeble degree and short range. Rutherford and Hahn, however, have shown that, by the disintegration of thorium, a very powerfully radio-active substance termed radio-thorium is produced. Dadourian and Boltwood, by different methods, have shown that while the thorium minerals are radio-active in proportion to their content of radio-thorium, pure thorium salts have only half of this radio-activity. This suggests the elimination of radio-thorium in the preparation of the pure thorium salts. Hahn, however, has found that thorium salts lose in radio-activity for three years, then regain their activity gradually up to a stable point. Hence, he concludes that the substance eliminated is an intermediate product between thorium and radio-thorium, which he terms meso-thorium. He has even recovered meso-thorium from the waste in the preparation of thorium proper. Two varieties of meso-thorium are described, the first losing half its power in five and one-half years, the second emitting beta and gamma rays and being destroyed in 6.2 hours. Both give birth to radio-thorium, which emits alpha rays and lives about two years, giving birth, in turn, to thorium X, the emanation from thorium, and to four types of thorium designated as A, B, C and D. Mesothorium, according to Soddy, is closely analogous to barium. In addition, minute quantities of radium are found in preparations of meso-thorium.

Mesothorium has been used therapeutically in plasters, capsules of glass or metal or by injection. It requires no filter and can be applied like radium. It costs only a quarter as much, but is reduced to half its efficiency in twenty years, whereas the life of radium is 1760 years. (Note—The calm way in which transmutation of metals is now mentioned, the genealogic terms applied to them and the very definite statements as to their "life" is somewhat disconcerting to one who studied physics and chemistry a few years ago.) Various authors are cited who have obtained marked amelioration of cancers, lupus, etc., by the use of meso-thorium, apparently by the stimulation of fibrous tissue, with new capillaries, which choke out the meoplastic cells.

Of all radio-active products of thorium, the most interesting is thorium X, of the fourth generation from the disintegration of thorium, and immediately preceding thorium emanation, as radium precedes radium emanation. Thorium X gains 10-20 per cent. in efficiency in the first day after its production, then loses about 17.5 per cent. a day till, by the third or fourth day, it possesses only 50 per cent. of its original power. Thorium X probably owes its activity to the nascence of thorium emanation, which dies very rapidly, in 53 seconds, giving place successively to thorium A, B, C and D. It thus results that it emanates soft alpha and beta rays, but, at the end of a short time, also gamma rays, in consequence of the formation of thorium D. Thorium X is supplied by Knöfler of Berlin in physiologic serum, 1 c.c. of which contains one hundred thousandth of a milligram of thorium X and represents an activity of a million electro-static units of Mache. This costs $2.50, but, unfortunately, loses about a fifth of its activity every day, so that its use is practical only in the vicinity of the place of manufacture, and for this reason most of the reports upon it are from German physicians. It has been employed subcutaneously, intravenously, by inhalation, drafts, baths, and by electrolytis introduction. Plesch and Karzog have shown by animal experiments (to which antivivisectionists should scarcely object) that about 12-18 per cent. is eliminated in the urine and fæces, about 80 per cent. being retained in the organism. Its fixation in the tissues occurs mainly in the medulla of bones. In 24 hours, 64 per cent. is found in such combination, the remainder in the intestine, liver and suprarenal capsules. Pappenheim and Plesch by administering lethal doses to rabbits have observed a rapid destruction of leucocytes and even a complete disappearance from the blood. The lymphocytes disappear first, then the uninuclears, eosinophiles and mast cells, in order, the multiformenuclears being the most resistant. The red cells show marked anisocytosis, but not degeneration and there are no blastocytes. At the beginning of the third day there are usually no polymorphic cells left and death generally occurs on the fourth day. Consecutive injections of sodium nucleinate do not succeed in restoring the leucocytes. Macroscopically, there are no hæmorrhages; the medulla of the bones is dark, the spleen black and atrophic.

Naturally, thorium X has been tried in leucocythæmia and with results, by numerous authors, surprisingly in accord with the theory. Plesch reduced the white cells from 190,000 to 4,186 in 15 days. Other symptoms were ameliorated and the spleen reduced in size. Acting on the principle of the opposite action of small doses, minute doses have been tried in pernicious anæmia with equally brilliant results, Plesch claiming a gain from 390,000 to 3,000,000 in 23 days.

While radium augments the activity of certain ferments, thorium X has no action on either digestive ferments nor bacteria. It has no action on respiration in the normal subject, but has a favorable action in pathologic modifications. It augments the cardiac diastole in cold-blooded animals. In warm-blooded animals, including man, it has little action on the blood pressure normally, but lowers states of high blood pressure, the effect lasting for several days. It augments nutritive exchanges, increasing both the consumption of oxygen and the elimination of carbon dioxid. In obesity, Plesch records a loss of 20 kilograms from 114, in 41 days. In gout, uric acid and purins are increased in the urine and the tophi diminish. Favorable results have been obtained in muscular and articular rheumatism, arthritis deformans and sclerodermia.

Caan and Czerny have employed thorium X in 106 cases of tumor, mostly cancer, but including 10 malignant lymphomata, with favorable influence in 40 per cent.

On the other hand, fatal results have been reported, as well as local burns from subcutaneous injections, probably due to the retention of radiothorium. Hence intravenous injections are to be preferred. Internal administration has caused diarrhœa and even hæmorrhages. Hence, a diet rich in waste, cathartics and enemata are recommended. Gastric and intestinal hæmornecrosis, inflammations of glandular organs and even "hæmorrhagic diatheses," including the disappearance of the bony medulla in one or two days and its replacement with a clot of blood, have been ascribed to too large doses. 3000-5000 electrostatic units of Mache is the maximum dose according to Plesch.

Recently, Caan has used a special chlorid of thorium, resulting from the manufacture of mesothorium, and hence including traces of this substance, in experimental carcinoma and sarcoma of rats and mice. After three or four injections, on alternate days, into the tumors, necrosis sets in, is followed by healthy granulations and a normal cicatricial tissue results in eight days.

(Note—We have translated this article almost in full, on account of its fascinating interest. Considered simply as a story, such an account surpasses Jules Verne and H. Rider Haggard. The play given the imagination in this and certain other phases of scientific literature is in marked contrast to the pragmatism of a few years ago. And, in addition, the most skeptic reader must admit the weight of authority of technically trained scientists and clinicians and must acknowledge at least the possibility that what he is tempted to consider mere dreams, may prove to be actual facts, fraught with the most wonderfully practical benefit to mankind.)

The following diagrammatic representation of these series of radio-active elements shows the order in which the products occur, their periods, and the rays which they give off in the process of their transmutation:

| | | |
|---|---|---|
| URANIUM 1<br>5,000,000,000 years<br>alpha rays | | |
| URANIUM 2<br>1,000,000 years (?)<br>alpha rays | | THORIUM<br>13,000,000,000 years<br>alpha rays |
| URANIUM X<br>24.6 days<br>beta, gamma rays | | MESOTHORIUM 1<br>5.5 years<br>Rayless |
| URANIUM Y<br>1.5 days<br>beta rays | ACTINIUM<br>Period unknown<br>Rayless | MESOTHORIUM 2<br>6.2 hours<br>beta, gamma rays |
| IONIUM<br>200,000 years (?)<br>alpha rays | RADIO ACTINIUM<br>19.5 days<br>alpha rays | RADIOTHORIUM<br>2 years<br>alpha rays |
| RADIUM<br>2,000 years<br>alpha rays | ACTINIUM X<br>10.2 days<br>alpha rays | THORIUM X<br>3.65 days<br>alpha rays |
| EMANATION (Niton)<br>3.86 days<br>alpha rays | ACT. EMANATION<br>3.9 seconds<br>alpha rays | TH. EMANATION<br>54 seconds<br>alpha rays |
| RADIUM A<br>3 minutes<br>alpha rays | ACTINIUM A<br>0.002 second<br>alpha rays | THORIUM A<br>0. 14 second<br>alpha rays |
| RADIUM B<br>26.8 minutes<br>beta, gamma rays | ACTINIUM B<br>36 minutes<br>beta rays | THORIUM B<br>10.6 hours<br>beta rays |
| RADIUM C<br>19.5 minutes<br>alpha, beta, gamma rays | ACTINIUM C<br>21 minutes<br>alpha rays | THORIUM C1<br>60 minutes<br>alpha rays |
| RADIUM D<br>16.5 years<br>beta rays | ACTINIUM D<br>4.71 minutes<br>alpha, beta, gamma rays | THORIUM C2<br>Period very short (?)<br>alpha rays |
| RADIUM E<br>5.0 days<br>beta, gamma rays | | THORIUM D<br>3. 1 minutes<br>beta, gamma rays |
| RADIUM F (polonium<br>136 days<br>alpha rays | | BISMUTH (?) |
| LEAD (?) | | |

C. H. VIOL, Radium, June, 1913.

